# Identifying Regulatory Posttranslational Modifications of PD-L1: A Focus on Monoubiquitinaton

**DOI:** 10.1016/j.neo.2017.02.006

**Published:** 2017-03-19

**Authors:** Henrick Horita, Andy Law, Soonjin Hong, Kim Middleton

**Affiliations:** R&D Department, Cytoskeleton Inc., Denver, CO, 80223, USA

## Abstract

A set of high-affinity, high-specificity posttranslational modification (PTM) enrichment tools was developed to generate an unbiased snapshot of four key PTM profiles (tyrosine phosphorylation, acetylation, ubiquitination, and SUMOylation 2/3) for the clinically important protein programmed cell death ligand 1 (PD-L1). The results showed that epidermal growth factor (EGF) treatment induced tyrosine phosphorylation, acetylation, and ubiquitination of PD-L1. Further characterization of EGF-induced PD-L1 ubiquitination revealed a significant increase in mono- and multiubiquitination of PD-L1 that occurred on glycosylated PD-L1. EGF induced mono- and multiubiquitination of PD-L1 preceded EGF-induced increases in PD-L1 protein levels. Chemical inhibitors of the EGFR pathway, gefitnib and SCH772984, suppressed PD-L1 mono- and multiubiquitination, and inhibition of the ubiquitin E1 activating enzyme, with the chemical inhibitor PYR41, was sufficient to block EGF-stimulated increases in PD-L1 protein levels. This study highlights the significance of identifying novel PTMs for PD-L1 and reveals potentially critical regulatory mechanisms that may be valuable therapeutic targets. In a broader context, this report validates an approach whereby one can gain insight into novel mechanisms of action by a simple and unbiased analysis of a PTM profile of potentially any endogenous protein of interest.

## Introduction

Programmed cell death ligand 1 (PD-L1) is a 33-kDa type I transmembrane protein that is comprised of an extracellular, N-terminal domain that interacts with receptor PD-1 expressed on Tcells to inhibit the immune response. PD-L1 is expressed in a wide range of cell types and tissues and has been shown to be upregulated in many of these cell and tissue types under inflammatory conditions [1]. Inhibition of the immune response through the PD-L1/PD-1 axis under normal physiological conditions helps maintain the balance between tolerance and autoimmunity; hence, PD-L1 is a key player in immune homeostasis. Pathologically, disruption of the PD-L1/PD-1 axis can lead to a host of immune-compromised diseases including lupus and arthritis [2].

Host immune suppression by cancer cells through cell surface expression of checkpoint inhibitors like PD-L1 is a key mechanism for cancer progression [3]. PD-L1 is overexpressed in many different cancers including breast, bladder, colon, melanoma, squamous cell carcinoma of the lung, and head and neck [1]. Although there has been a long-standing interest in activating the patient's immune system to treat cancer, there have been few viable therapies [4]. Recently, two critical clinical trials on inhibitors of the PD-L1/PD-1 axis validated the premise that regulating immune checkpoint inhibitors was an effective cancer therapy [5], [6]. Several antibody-based drugs targeting the PD-L1/PD-1 axis have now been approved by the FDA and are showing great promise in the clinic [7]; however, it is currently unclear as to why only some PD-L1–positive tumors respond to PD-L1/PD-1 axis inhibition [8]. A better understanding of the mechanisms regulating PD-L1 function may help design better biomarkers and/or more efficacious therapeutic approaches.

A large body of work exists describing the transcriptional and posttranscriptional regulation of PD-L1 expression [9]. In contrast, it is difficult to find reports regarding posttranslational regulation of this protein, which is surprising given PD-L1’s clinical relevance and the recognized importance of posttranslational modifications (PTMs) in protein regulation in general [10]. A very recent report was published by Li et al. in *Nature Communications* describing the regulation of PD-L1 via both polyubiquitination and glycosylation PTMs [11].

Several PTMs have been studied in great detail; of these, serine/threonine phosphorylation, tyrosine-phosphorylation (pY), acetylation (Ac), ubiquitination (Ub), and SUMOylation (SUMO 2/3) have been shown to be key regulators in almost all cellular processes, including signal transduction, protein expression, stability and localization, and cellular immunity [12], [13], [14], [15]. In this study, a set of high-affinity, high-specificity PTM enrichment tools was utilized to generate an unbiased snapshot of four key PTM profiles (pY, Ac, Ub, and SUMO 2/3) for the clinically important protein PD-L1. The aim of this study was to utilize this newly developed toolkit to gain mechanistic insight about potential PTMs that regulate PD-L1 while also validating that these tools are an effective method to rapidly obtain information on the endogenous PTM regulation of any target protein.

## Experimental Procedures

### Cell Culture and Reagents

A431 cells were grown in DMEM media (ATCC, VA) supplemented with 10% FBS (Atlas Biologicals, CO) and penicillin/streptomycin (ThermoFisher, MA). Trypsin/EDTA was obtained from Gibco (ThermoFisher, MA). Unless otherwise noted, chemicals were obtained from Sigma (Sigma, MO). Human epidermal growth factor (EGF) was obtained from Cytoskeleton, Inc. (Cytoskeleton, CO). For EGF stimulation experiments, A431 cells were serum restricted for 24 hours with serum-free DMEM to synchronize the cells. Cells were then treated with 33 ng/ml of EGF for 15 minutes or 1, 2, and 4 hours in individual 15-cm dishes (Corning, NY) followed by subsequent lysis with BlastR lysis buffer (Cytoskeleton, CO). Gefitnib, SCH772984, and PYR-41 were obtained from Selleckchem (Selleckchem, TX). For chemical inhibition experiments, synchronized A431 cells were pretreated with 0.5 μM gefitnib, 2 μM SCH772984, or 100 μM PYR-41 for 30 minutes at 37°C prior to EGF treatment for 1 hour followed by lysis with BlastR lysis buffer.

### Western Immunoblotting

A431 cells were lysed with ice-cold BlastR lysis buffer containing a cocktail of NEM, TSA, Na_3_VO_4_, and protease inhibitors (Cytoskeleton, CO). DNA was removed by passing the lysate through the BlastR filter system (Cytoskeleton, CO). BlastR lysis buffer is a complete cell lysis reagent that is comprised of a proprietary mixture of detergents, salts, and other buffer additives. After dilution with BlastR dilution buffer, protein concentrations were determined with Precision Red Advanced protein reagent (Cytoskeleton, CO) and measured at 600 nm OD. Protein lysate samples were separated using Tris-glycine SDS-polyacrylamide gel electrophoresis (ThermoFisher, MA) and transferred to Immobilon-P membranes (Millipore, MA). Membranes were blocked for 30 minutes at room temperature in Tris-buffered saline (10 mM Tris-HCl, pH 8.0, 150 mM NaCl) containing 0.05% Tween-20 (TTBS) and 5% milk (Thrive Life, UT) and then incubated with 0% to 2.5% milk in TTBS solution containing primary antibodies for 1 to 3 hours at room temperature (RT). Membranes were washed in TTBS 3 ×10 minutes prior to secondary antibody for 1 hour at RT. Bound antibodies were visualized with horseradish peroxidase–coupled secondary antibodies and chemiluminescent reagent (Cytoskeleton, CO) according to the manufacturer's directions. Antibodies used were as follows: P-EGFR ab40815 (Abcam, MA), PD-L1 ab213524 (Abcam, MA) or PD-L1 13,684 (Cell Signaling, MA), anti-ubiquitin-HRP AUB01-HRP (Cytoskeleton, CO), tubulin ATN02 (Cytoskeleton, CO), HRP-anti-sheep secondary (Cytoskeleton, CO), and HRP-anti-rabbit secondary (Jackson ImmunoResearch, PA). Changes were quantitated by densitometry using Image J software (rsb.info.nih.gov).

### Immunoprecipitation Assay

After obtaining A431 lysate as described above in the western immunoblotting section, samples were immunoprecipitated using pY, Ub, Ac, and SUMO 2/3 PTM identification, Signal-Seeker kits, with equal protein concentration and IP volumes according to the manufacturer's protocol (Cytoskeleton, CO). The appropriate amount of pY (APY03), ub (UBA01), SUMO 2/3 (ASM24), Ac (AAC01), IgG beads (CIG01), or control beads (CUB01) was added to the respective samples for 1 to 2 hours at 4°C on an end-over-end tumbler. After incubation, the affinity beads from each sample were pelleted and washed 3× with BlastR wash buffer. Bound proteins were eluted using the elution buffer and spin columns in the Signal-Seeker kits, and PTM modified target proteins were detected by western immunoblotting.

### Deglycosylation Assay

A431 cells were lysed with ice-cold BlastR lysis buffer containing a cocktail of NEM, TSA, Na_3_VO_4_, and protease inhibitors. IP was performed on lysate using ubiquitin affinity beads (UBA01) or control beads (CUB02). Ubiquitinated proteins were eluted with an alternative elution buffer (50 mM Tris pH 8.0, 0.2% SDS, 0.1% Tween-20) for 15 minutes at 25°C. Immunoprecipitated and WCL samples were then deglycosylated using PNGase F from NEB according to the manufacturer's instructions (NEB, MA) with the following modifications: PNGase F was used at 0.2 μl per reaction with an incubation time of 30 minutes at 37°C. Additionally, during the SDS neutralization step, additional NP-40 was used to account for the additional 0.2% SDS in the elution step. Finally, samples were run on SDS-PAGE, transferred to PVDF, and analyzed by western immunoblotting.

## Results and Discussions

### EGF Stimulation Induces Both PD-L1 Protein Levels and PD-L1 PTMs

**Figure 1:**
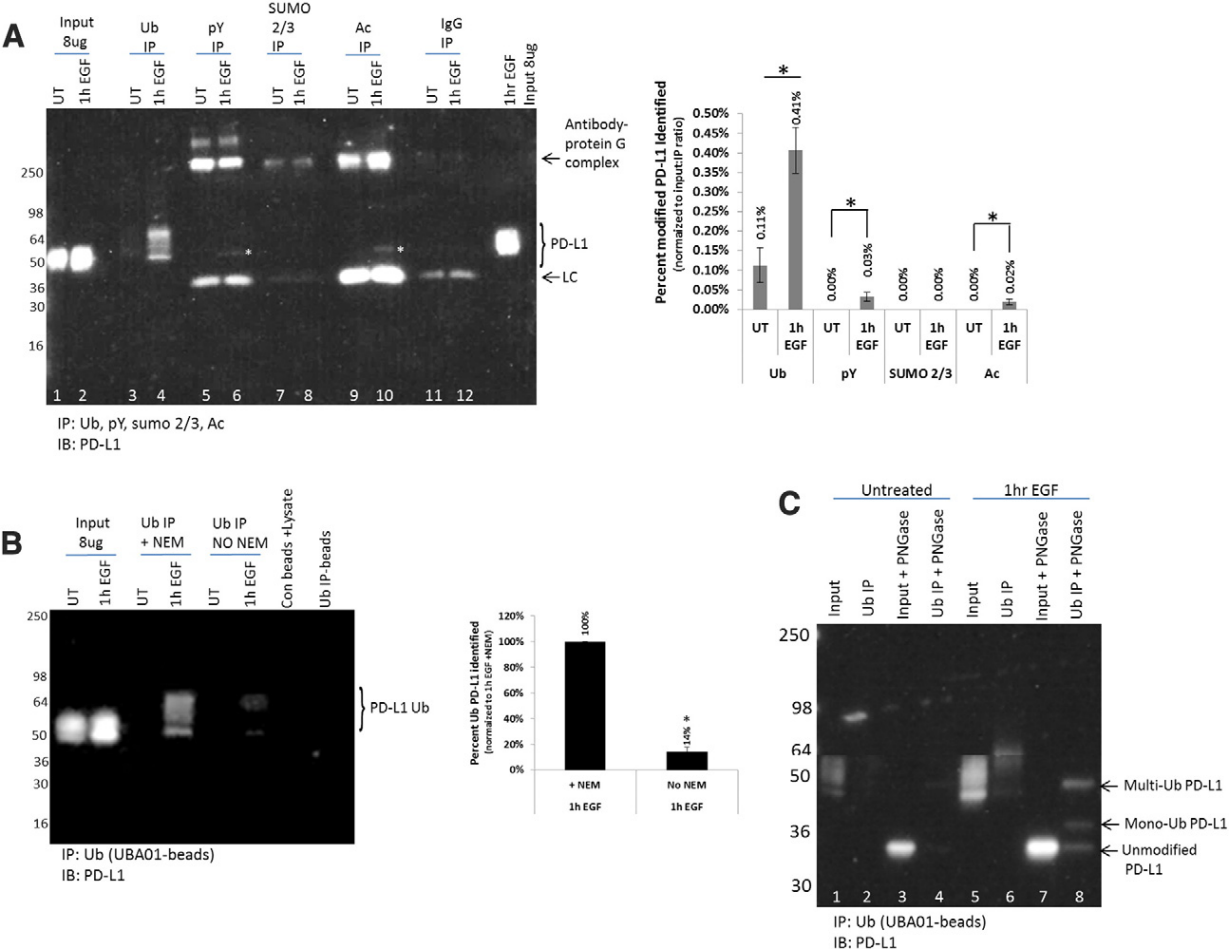
Detection of EGF-induced PTMs for PD-L1. (A) Serum-restricted A431 cells were either unstimulated (UT) or stimulated with EGF for 1 hour prior to lysis with BlastR lysis buffer. WCL was analyzed for PD-L1 levels (lanes 1 and 2). Ubiquitin binding beads (UBA01) were used to IP ubiquitinated proteins (lanes 3 and 4). Phosphotyrosine binding beads (APY03) were used to IP tyrosine-phosphorylated proteins (lanes 5 and 6). SUMO 2/3 binding beads (ASM24) were used to IP SUMOylated 2/3 proteins (lanes 7 and 8). Acetyl lysine binding beads (AAC01) were used to IP acetylated proteins (lanes 9 and 10). IgG binding control beads were used to IP nonspecific binding proteins (lanes 11 and 12). Samples were separated by SDS-PAGE and analyzed by Western blot using a PD-L1 antibody. Left: a representative blot from *N* ≥ 3 independent experiments. White asterisks were used to highlight PD-L1 pY and Ac protein bands. Right: percent change in densitometry measurements that were normalized to respective inputs as well as input:IP ratio ± S.E.M. *P < .05. (B) A431 cells were harvested with BlastR lysis buffer with or without NEM. Lysates were incubated with UBA01 ubiquitin binding beads and analyzed for ubiquitinated PD-L1. Left: a representative blot from *N* ≥ 3 independent experiments. Right: percent change in densitometry measurements ± S.E.M. *P < .05. (C) Serum-restricted A431 cells were either UT or stimulated with EGF 1 hour prior to lysis with BlastR lysis buffer. Lysates were incubated with UBA01 ubiquitin binding beads. After elution with modified elution buffer, samples were incubated with or without PNGaseF. Samples were separated by SDS-PAGE and analyzed by Western blot for PD-L1. Shown is a representative western blot from *N* ≥ 3 independent experiments.

Several studies have linked increased epidermal growth factor receptor (EGFR) signaling, either through mutation or ligand-induced activation, to increased PD-L1 expression, which has been shown to contribute to the immune evasion of EGFR-driven cancers [16], [17]. Data in Figure 1*A*, *lanes 1 and 2*, recapitulated previously published results that PD-L1 protein levels increased in response to EGF stimulation in the A431 cell line [11], thus providing a rationale to utilize this model system to further examine changes in PD-L1 abundance as well as potential changes in the PD-L1 PTM profile. To examine the PTM profile of PD-L1 in response to EGF stimulation, affinity enrichment of pY, Ac, Ub, and SUMO 2/3 modified proteins was performed on A431 cell lysate. Immunoprecipitated samples were separated by SDS-PAGE and analyzed by western blot detection of PD-L1. EGF stimulation resulted in a small but significant induction of PD-L1 tyrosine phosphorylation and acetylation (Figure 1*A*, *lanes 5,6,9,and10*); conversely, neither untreated nor EGF-treated A431 cells showed any evidence of SUMO 2/3 modification of PD-L1 (Figure 1*A*, *lanes 7 and 8*). Most interestingly, a marked increase in PD-L1 Ub was detected in response to EGF stimulation (Figure 1*A*, *lanes 3 and 4*).

Densitometric quantitation of ubiquitinated PD-L1 normalized to input:immunoprecipitation (IP) ratio suggests that an estimated .41% of the total PD-L1 pool is ubiquitinated in response to EGF (Figure 1*A*, densitometry). This is a minimal estimate of the fractional stoichiometry as the absolute efficiency of ubiquitin enrichment for PD-L1 Ub has not been determined. In this regard, it is known that the vast majority of endogenous PTMs is substoichiometric and require an enrichment step for detection [18]. The enrichment strategy and detection method utilized here to identify PD-L1 ubiquitination were successful; in contrast, identification of PD-L1 ubiquitination by mass spectrometry was unsuccessful (data not shown). Further investigation comparing the sensitivity of these two methods is warranted.

To confirm that the ubiquitinated PD-L1 signal was truly ubiquitinated, cell lysates were processed from untreated and EGF-treated A431 cells in the absence of the pan de-ubiquitinase (DUB) inhibitor *N*-ethylmaleimide (NEM). The presence of NEM protects ubiquitinated species from rapid de-ubiquitination by DUBs during lysate processing. The results in Figure 1*B* show that cell lysates lacking DUB inhibitor have more than an 80% decrease in ubiquitinated PD-L1 signal and demonstrates that the ubiquitinated PD-L1 signal is dependent on protein Ub and is not likely a false-positive signal. The detection of endogenous levels of PD-L1 Ub that is dynamically regulated by EGF suggested physiological relevance of PD-L1 Ub and warranted further investigation.

### EGF Induces PD-L1 Mono- and Multiubiquitination

The EGF-induced Ub signal for PD-L1 appears to represent a pattern of mono- and multi-/di- (here forward termed multi-) Ub as indicated by the discreet bands of 55 to >64 kDa (Figure 1, *A* and *B*), which are representative of a molecular weight shift of 1 to 2 ubiquitin modifications. To gain further insight into the type of Ub occurring on PD-L1, IP of Ub was performed with affinity reagents that are known to bind monoubiquitinated proteins (FK2 antibody) [19], as well as an affinity reagent that binds to monoubiquitin with low affinity (UbiQ) (unpublished finding). These results were compared to findings from the ubiquitin affinity beads (UBA01) used throughout this study, which are an affinity matrix composed of a proprietary mixture of ubiquitin binding domains that are capable of capturing mono- and polyubiquitinated proteins. The EGF-induced PD-L1 Ub pattern in the 55- to >64-kDa region was similar between the UBA01 and FK2 IP (Figure S1). Importantly, this same region is almost completely void of signal in the EGF-treated UbiQ IP lane. Collectively, these data suggest that the 55- to >64-kDa bands likely represent mono- and multiubiquitinated PD-L1 (Figure S1).

The smeared banding of PD-L1 in both the ubiquitin IP and the whole cell lysate (WCL) made it difficult to decipher which band corresponded to the monoubiquitinated PD-L1. A previous study reported that the smeared PD-L1 signal observed in the WCL was due to extensive glycosylation of PD-L1 [11]. To remove glycosylation of PD-L1, they performed *in vitro* deglycosylation with PNGase F, which resulted in a single unmodified PD-L1 protein band at 33 kDa. The *in vitro* deglycosylation method was utilized in this study and resulted in a collapse of the WCL bands between 45 and 55 kDa to a single species right below the 36-kDa molecular weight marker (Figure 1*C*, *lane 5 vs 7*), which was very similar to the previously reported findings. The *in vitro* deglycosylation step was also performed on ubiquitin immunoprecipitated samples from EGF-treated cell lysate, which resulted in a collapse of the ubiquitinated PD-L1, 55- to >64-kDa smear, to three discrete bands that migrated below the 36-kDa maker, between the 36-kDa and 50-kDa marker, and right at the 50-kDa marker (Figure 1*C*, *lane 6 vs 8*). These size changes of roughly 8 kDa between bands correlate with the molecular weight of ubiquitin, providing further evidence that EGF stimulation induces a mono- and multi-Ub of PD-L1. The exact PD-L1 Ub site has not been defined; however, the UbPred prediction software [20] identified two lysine residues in PD-L1 (K178, k281) with high confidence as potential Ub sites. Future studies are warranted to identify where PD-L1 is mono- and multiubiquitinated.

### EGF-Stimulated PD-L1 Monoubiquitination Occurs Prior to Enhancement of Total PD-L1 Protein Levels

**Figure 2:**
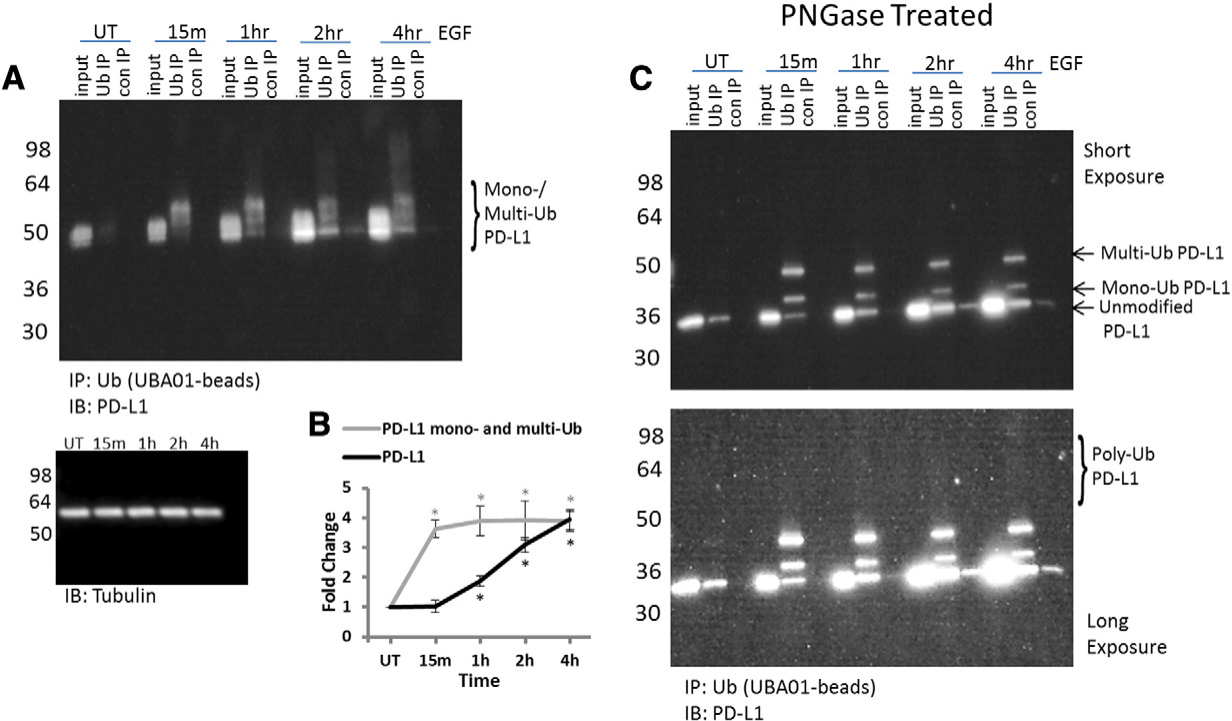
Defining temporal changes in PD-L1 total protein relative to PD-L1 ubiquitination. (A) Serum-restricted A431 cells were stimulated with EGF for the given time period. Lysates were incubated with UBA01 ubiquitin binding beads. WCL and Ub samples were separated by SDS-PAGE and analyzed by western blot for PD-L1. Shown is a representative Western blot from *N* ≥ 3 independent experiments. (B) Quantification of densitometric analysis of total PD-L1 and ubiquitinated PD-L1 protein changes in response to EGF stimulation. *P < .05. (C) Serum-restricted A431 cells were stimulated with EGF for the given time period. Lysates were incubated with UBA01 ubiquitin binding beads. After elution with modified elution buffer, samples were incubated with or without PNGaseF. WCL and Ub samples were separated by SDS-PAGE and analyzed by western blot for PD-L1. Shown is a representative western blot from *N* ≥ 3 independent experiments with either a short exposure or a long exposure.

Previously published data on Ub of PD-L1 identified poly-Ub as a mechanism for proteasomal-dependent PD-L1 downregulation [11]. In contrast, data from this study showed an enhancement of PD-L1 mono- and multi-Ub as well as an increase in total PD-L1 protein levels in response to EGF stimulation (Figure 1*A*). The increase in PD-L1 protein levels in response to EGF corroborates previously published findings by several groups [16], [17]. Based on these findings, the question arose as to whether PD-L1 mono-Ub and multi-Ub also downregulate PD-L1 similarly to PD-L1 poly-Ub. To begin addressing this question, both PD-L1 protein levels and ubiquitinated PD-L1 levels were analyzed from a time course of EGF-treated A431 cells. As expected, PD-L1 protein levels increased in EGF-treated A431 cells over the 4-hour time course of this experiment with a distinct increase beginning at 1 hour (Figure 2*A*). Surprisingly, a rapid increase in PD-L1 mono-Ub at 15 minutes consistently preceded the increase in PD-L1 levels (Figure 2*A*). Densitometric analysis highlighted the significant difference between the change in mono-Ub versus total Ub at 15 minutes with a measured three-fold increase in PD-L1 mono-Ub but no measureable increase in total PD-L1 protein levels (Figure 2*B*). The mono- and multi-Ub (50->64 kDa) bands of PD-L1 are the predominant species throughout this time course, particularly at the 15-minute time point (Figure 2*A*). These findings were clarified even further by deglycosylating the WCL and ubiquitinated PD-L1 samples, which resulted in two distinct bands corresponding to a mono- and multi-Ub molecular weight shift at the 15-minute time point without any significant increase in total PD-L1 (Figure 2*C*). The fact that mono-Ub precedes the increase in PD-L1 levels suggests that PD-L1 mono-Ub is not simply a consequence of increased PD-L1 expression and raises the intriguing possibility that mono-Ub is required for the EGF-driven enhancement of PD-L1 protein levels. Later time points, 1 to 4 hours, also show clear evidence of polyubiquitinated species which are indicated by the nondiscreet, smeary signal from 80 to 200 kDa (Figure 2, *A* and *C*). Whether or not the same PD-L1 molecule is both mono- and polyubiquitinated is not known, but based on the information that glycosylation and poly-Ub are mutually exclusive, one may hypothesize that the mono-Ub and poly-Ub are occurring on different PD-L1 molecules. Why EGF would activate both mono- and poly-Ub mechanisms is also unknown, but it may suggest that there is a constant flux or turnover of PD-L1 molecules that may be important for EGF-regulated cellular function.

### Chemical Inhibitors of the EGFR Pathway Regulates PD-L1 Mono- and Multiubiquitination and Total PD-L1 Protein Levels

**Figure 3:**
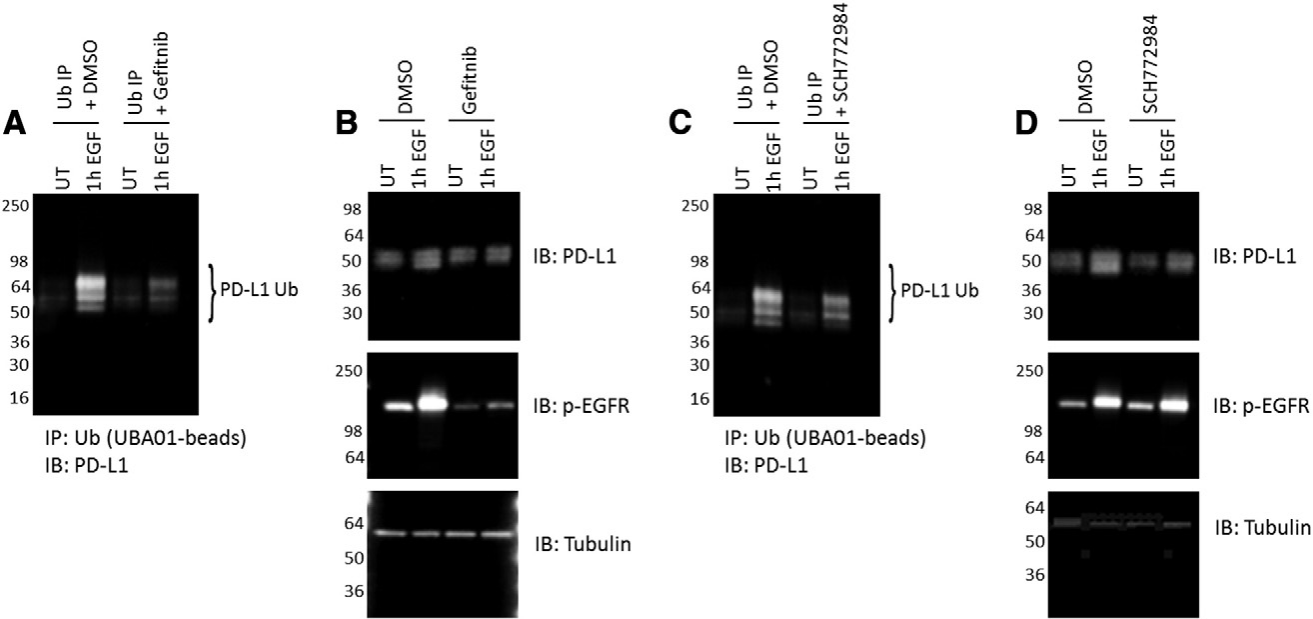
Defining EGF-dependent regulation of PD-L1 mono- and multiubiquitination using EGFR pathway chemical inhibitors. (A) Serum-restricted A431 cells were pretreated with gefitnib for 30 minutes prior to stimulation with EGF for 1 hour. Cells were lysed and incubated with UBA01 ubiquitin binding beads. Samples were separated by SDS-PAGE and analyzed by western blot for PD-L1. (B) WCL was analyzed for total PD-L1 levels and activated EGFR levels (p-EGFR). Tubulin was used as a loading control. (C) Serum-restricted A431 cells were pretreated with SCH772984 for 30 minutes prior to stimulation with EGF for 1hour. Cells were lysed and incubated with UBA01 ubiquitin binding beads. Samples were separated by SDS-PAGE and analyzed by western blot for PD-L1. (D) WCL was analyzed for total PD-L1 levels and activated EGFR levels (p-EGFR). Tubulin was used as a loading control. For all experiments, shown are representative western blots from *N* ≥ 3 independent experiments.

To gain further insight into the relationship between PD-L1 mono- and multi-Ub and PD-L1 protein levels, inhibitors of the EGF signaling pathway that are known to block EGF-induced PD-L1 expression [16] were used to determine if EGF signaling is required to increase PD-L1 mono- and multi-Ub. Treatment of A431 cells with 0.5 μM gefitnib, a selective inhibitor of the EGFR tyrosine kinase signaling domain, effectively blocked EGF activation as determined by phosphorylation of EGFR (Figure 3*B*). Effective inhibition of EGFR activation and signaling significantly decreased PD-L1 mono- and multi-Ub (Figure 3*A*) and prevented the accumulation of PD-L1 protein (Figure 3*B*). Similar results on PD-L1 protein levels and mono- and multi-ubiquitination were observed with the specific Erk inhibitor SCH772984 while having no effect on upstream activation of EGFR (Figure 3, *C* and *D*), thus suggesting that EGF-regulated Ub of PD-L1 is mediated at least in part through ERK signaling. Together, these results indicated that the inhibition of total PD-L1 accumulation in response to EGF stimulation is proportional to the reduction in PD-L1 mono- and multi-Ub and support the hypothesis that EGF-induced PD-L1 Ub is intricately intertwined with EGF-mediated increase in PD-L1 protein levels. Although this current study focused specifically on EGF regulation of PD-L1 Ub, it will be interesting to determine if alternative growth factors and cytokines (i.e., interferon gamma) that are known to enhance PD-L1 expression also regulate PD-L1 Ub. Conversely, utilization of non-EGF pathway inhibitors may shed light on the specificity of the regulation of PD-L1 Ub by EGF.

### Chemical Inhibition of the Ubiquitin E1 Activating Enzyme Regulates PD-L1 Mono- and Multiubiquitination and Total PD-L1 Protein Levels

**Figure 4:**
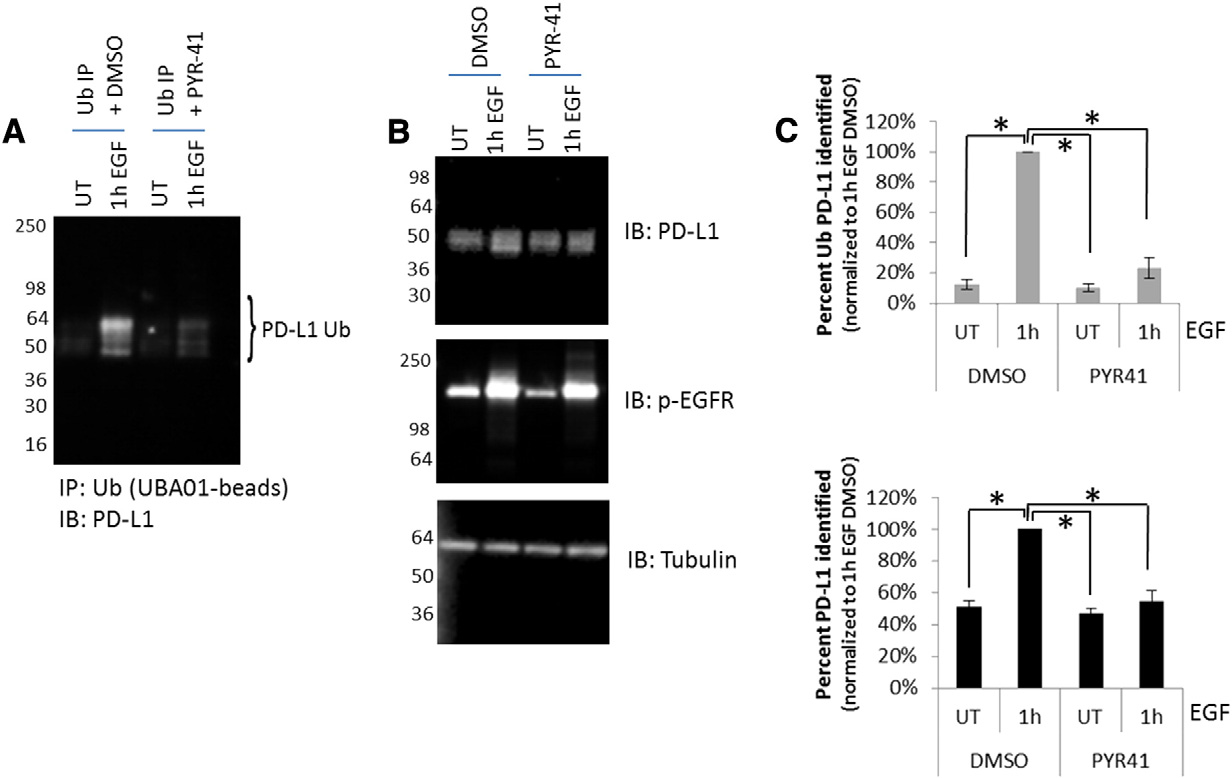
Defining the role of PD-L1 mono- and multiubiquitination on total PD-L1 protein levels using ubiquitin E1 activating enzyme chemical inhibitors. (A) Serum-restricted A431 cells were pretreated with PYR-41 for 30 minutes prior to stimulation with EGF for 1 hour. Cells were lysed and incubated with UBA01 ubiquitin binding beads. Samples were separated by SDS-PAGE and analyzed by western blot for PD-L1. (B) WCL was analyzed for total PD-L1 levels and activated EGFR levels (p-EGFR). Tubulin was used as a loading control. (C) Quantification of densitometric analysis of ubiquitinated PD-L1 (upper panel) and total PD-L1 (lower panel) changes in response to PYR-41 treatment prior to EGF stimulation. *P < .05. For all experiments, shown are representative western blots from *N* ≥ 3 independent experiments.

To further explore the possibility that PD-L1 mono-Ub and multi-Ub regulate total PD-L1 protein levels, we wanted to inhibit the Ub of PD-L1 through direct interference with the ubiquitin machinery. The E3 ligase that regulates PD-L1 mono- and multi-Ub is unknown, but since there is only one E1 ubiquitin activating enzyme in humans that regulates the entire Ub machinery, inhibiting E1 function is a valid tool to examine ubiquitination. The E1 specific inhibitor PYR-41 has been shown to effectively block protein Ub [21]. Cells treated with 100 μM PYR-41 or DMSO control for 30 minutes prior to EGF treatment for 1 hour were examined for EGFR activation, PD-L1 levels, and PD-L1 mono- and multi-Ub. Treatment with PYR-41 significantly reduced the level of PD-L1 mono- and multi-Ub (Figure 4, *A* and *C*) while having no effect on the relative activation of the EGFR as determined by phosphorylation of EGFR (Figure 4*B*). Quantitation of total PD-L1 revealed that the EGF-mediated increase of PD-L1 was abolished in PYR-41–treated cells (Figure 4, *B* and *C*). Although obvious caveats exist with interpretation of these data against a backdrop of total inhibition of the global ubiquitin machinery, taken as a whole, the data are consistent with the hypothesis that mono-Ub of PD-L1 can operate as a mechanism to regulate EGF/EGFR-enhanced PD-L1 protein levels. Although utilizing an E1 chemical inhibitor to block Ub may not provide the desired therapeutic specificity, identification of the E3 ligase that regulates mono-Ub of PD-L1 may have promise as a therapeutic target.

### Targeting PD-L1 PTMs May Be a Novel Therapeutic Approach to Targeting the PD-L1/PD-1 Axis

**Figure 5:**
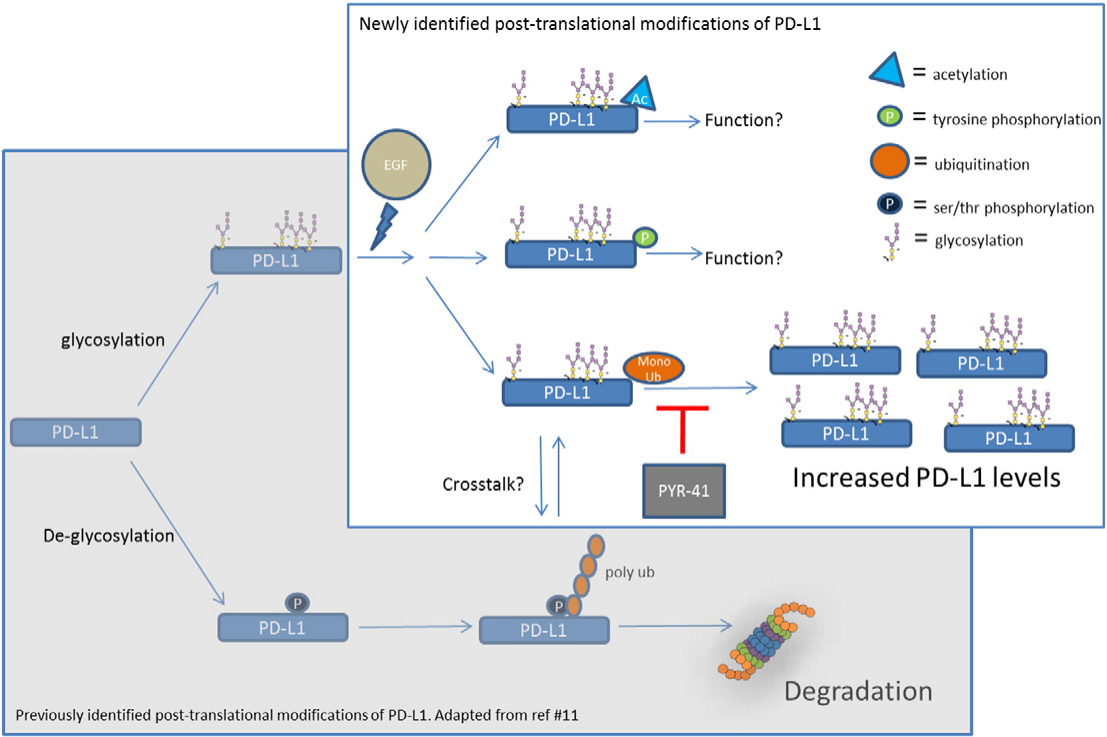
Model: Profile of PD-L1 post-translational modifications and their roles in regulating PD-L1 protein levels.

In this study, PTMs of PD-L1 in response to EGF stimulation were analyzed through a nonbiased snapshot, and three novel PTMs of PD-L1 were identified, specifically, tyrosine phosphorylation, acetylation, and mono- and multi-Ub. An important role for PD-L1 mono- and multi-Ub was elucidated, which was to facilitate EGF-induced PD-L1 protein levels. Our study combined with the work by Li et al. [11] supports the proposal that Ub is a key regulator of PD-L1 proteostasis and suggests that Ub of PD-L1 may act to either stabilize or destabilize PD-L1 possibly via a balance between mono- and multi- versus poly-Ub (Figure 5). This type of observation showing opposing roles for mono- versus poly-Ub is not without precedent. It has been reported that LIM-domain-binding 1 protein (LDB1) is regulated by a balance between mono- and poly-Ub in which substoichiometric levels of monoubiquitinated LDB1 serve to stabilize the total LDB1 population whereas polyubiquination of LDB1 leads to proteasomal degradation [22]. In these studies, it was proposed that mono-Ub protected LDB1 from poly-Ub and subsequent degradation, although the mechanism of how this works is currently unclear. As many cancers, in particular the EGFR-driven cancers, escape immune detection through PD-L1 expression, the link between mono-Ub and protein expression levels may open the door to novel cancer therapies.

As the focus of these studies was PD-L1 Ub, no additional information regarding the role for PD-L1 tyrosine phosphorylation or acetylation was uncovered. For example, whether they may regulate PD-L1 expression, function, or interaction with target proteins like the PD-1 receptor was not investigated. The signaling mechanisms that regulate the dynamics of these newly identified PD-L1 PTMs are still unknown, and it will be interesting to further dissect why EGF signaling activates all three PTMs as well as whether cross-talk or cross-regulatory mechanisms exist between these three PTMs and other PTMs like poly-Ub. These findings not only have implications for basic PD-L1 biology but may also aid in the design of more efficacious PD-L1/PD-1 drugs and/or diagnostic markers.

The following are the supplementary data related to this article.

Supplemental Figure S1 Characterization of PD-L1 mono- and multiubiquitination. (A). Serum-restricted A431 cells were either unstimulated or stimulated with EGF 1 hour prior to lysis with BlastR lysis buffer. Samples were immunoprecipitated with ubiquitin binding beads (UBA01), UbiQ poly-ubiquitin preferential binding beads, or FK2 antibody-based mono- and polyubiquitin binding beads. Samples were separated by SDS-PAGE and analyzed by Western blot for PD-L1. Shown is a representative Western blot from *N* ≥ 3 independent experiments. Supplemental Figure S1

## Author Contributions

H.H. conceived and performed experiments, and wrote the manuscript. A.L. and S.H. provided reagents, expertise, and feedback. K.M. conceived experiments, wrote the manuscript, provided expertise and feedback, and secured funding.
