# Supplementary material for: Identifying Regulatory Posttranslational Modifications of PD-L1: A Focus on Monoubiquitinaton
Source: Neoplasia. 2017 Mar 19;19(4):346–53. doi: 10.1016/j.neo.2017.02.006 (PMC5358937; doi:10.1016/j.neo.2017.02.006)
Supplement: Supplemental Figure S1 — Characterization of PD-L1 mono- and multiubiquitination. (A). Serum-restricted A431 cells were either unstimulated or stimulated with EGF 1 hour prior to lysis with BlastR lysis buffer. Samples were immunoprecipitated with ubiquitin binding beads (UBA01), UbiQ poly-ubiquitin preferential binding beads, or FK2 antibody-based mono- and polyubiquitin binding beads. Samples were separated by SDS-PAGE and analyzed by Western blot for PD-L1. Shown is a representative Western blot from N ≥ 3 independent experiments. [file mmc1.docx]

***
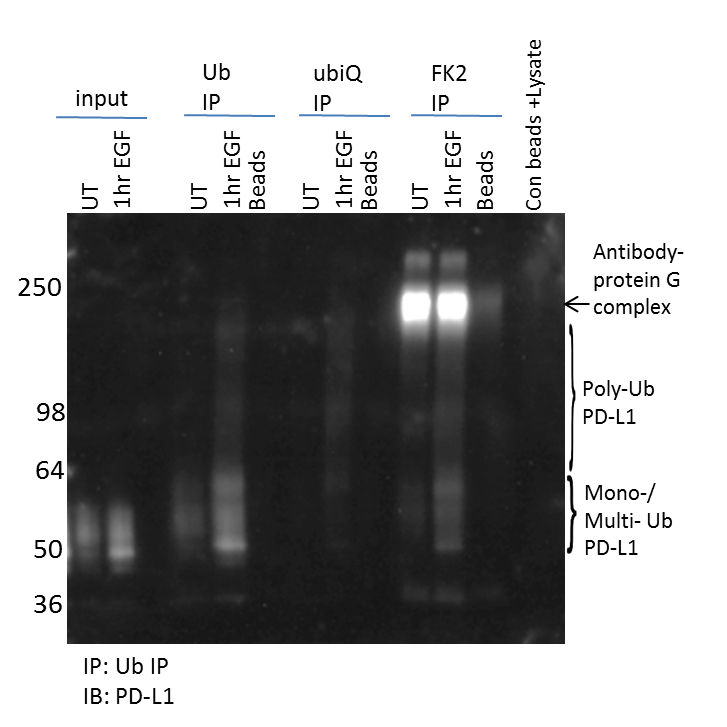
***

***Supplemental Figure 1. Characterization of PD-L1 mono- and multi-ubiquitination.*** (A). Serum restricted A431 cells were either unstimulated or stimulated with EGF one hour prior to lysis with BlastR lysis buffer. Samples were immunoprecipitated with ubiquitin binding beads (UBA01), UbiQ poly-ubiquitin preferential binding beads, or FK2 antibody based mono- and poly-ubiquitin binding beads. Samples were separated by SDS-PAGE and analyzed by western blot for PD-L1. Shown is a representative western from N≥3 independent experiments.
